# New Three-Dimensional Porous Electrode Concept: Vertically-Aligned Carbon Nanotubes Directly Grown on Embroidered Copper Structures

**DOI:** 10.3390/nano7120438

**Published:** 2017-12-11

**Authors:** Noemí Aguiló-Aguayo, Roger Amade, Shahzad Hussain, Enric Bertran, Thomas Bechtold

**Affiliations:** 1 Research Institute of Textile Chemistry and Textile Physics, University of Innsbruck, Hoechsterstrasse 73, 6850 Dornbirn, Austria; thomas.bechtold@uibk.ac.at; 2 FEMAN Group, Departament de Física Aplicada, Universitat de Barcelona, c/Martí i Franquès 1, 08028 Barcelona, Catalonia, Spain; r.amade@ub.edu (R.A.); sha.awan@hotmail.com (S.H.); ebertran@ub.edu (E.B.); 3 Institute of Nanoscience and Nanotechnology (IN2UB), Universitat de Barcelona, c/Martí i Franquès 1, 08028 Barcelona, Catalonia, Spain

**Keywords:** 3D porous electrodes, carbon nanotubes, embroidered current collectors, anode, plasma

## Abstract

New three-dimensional (3D) porous electrode concepts are required to overcome limitations in Li-ion batteries in terms of morphology (e.g., shapes, dimensions), mechanical stability (e.g., flexibility, high electroactive mass loadings), and electrochemical performance (e.g., low volumetric energy densities and rate capabilities). Here a new electrode concept is introduced based on the direct growth of vertically-aligned carbon nanotubes (VA-CNTs) on embroidered Cu current collectors. The direct growth of VA-CNTs was achieved by plasma-enhanced chemical vapor deposition (PECVD), and there was no application of any post-treatment or cleaning procedure. The electrochemical behavior of the as-grown VA-CNTs was analyzed by charge/discharge cycles at different specific currents and with electrochemical impedance spectroscopy (EIS) measurements. The results were compared with values found in the literature. The as-grown VA-CNTs exhibit higher specific capacities than graphite and pristine VA-CNTs found in the literature. This together with the possibilities that the Cu embroidered structures offer in terms of specific surface area, total surface area, and designs provide a breakthrough in new 3D electrode concepts.

## 1. Introduction

New concepts are required to increase the volumetric energy density and rate capabilities of lithium-ion (Li-ion) batteries. Some progress has already been made by replacing the metal foils, commonly used as current collectors, by three-dimensional structures, such as metal foams [1] and embroidered structures [2]. They were shown to support higher mass loadings than their two-dimensional (2D) metal foils counterparts. Another strategy to increase the usable capacity, and therefore the energy density, is to produce free-standing electrodes, which do not have a current collector [3,4]. However, they exhibit larger resistances and lower mechanical stability than conventional electrodes containing a current collector.

Graphite is commonly used as the electroactive material in the anodes of Li-ion batteries, and has a theoretical capacity of 372 mAh·g^−1^. In contrast, carbon nanotubes (CNTs) exhibit specific capacities of 300–1000 mAh·g^−1^, depending on their morphological characteristics [5,6]. This can be further improved by post-treatments. For instance, introducing defects or removing the metal caps (catalyst particles that initiate the growth of the CNTs) can reduce the energy barrier and facilitate Li^+^ diffusion into the inner core of the CNTs as well as their adsorption on the walls [7]. Another strategy to increase the specific capacity involves alloying/de-alloying with metal ions such as silicon or tin [8], as well as by conversion reactions with transition metal compounds such as iron, cobalt, or manganese [9,10].

Further advantages of CNTs are strong chemical resistance, high mechanical strength, and high electronic and thermal conductivity, which are attributes for energy storage systems as well as other applications, such as flexible electronics and solar energy [11,12].

One method of generating CNTs is through plasma-enhanced chemical vapor deposition (PECVD), which allows the formation of vertically aligned carbon nanotubes (VA-CNTs). VA-CNTs produced with PECVD are individual, free-standing vertical structures instead of the spaghetti-like or ensemble towers obtained by other techniques such as chemical vapor deposition (CVD) [13]. VA-CNTs exhibit greater rate capability than non-aligned CNT configurations due to their higher lithium-ion access, and higher electrical and thermal conductivity along the tubular axis [7,14,15,16].

The production of VA-CNTs on metallic current collectors is not immediate. The metal catalyst, which acts as a seed and is required for CNT growth, diffuses inside the metal substrate, limiting the CNT production. To avoid this, intermediate layers between the catalyst and the metal substrate are deposited [17]. In addition, the control of the catalyst diffusion plays an important role in the morphological characteristics and growth of VA-CNTs [18].

In this work, we investigated the use of embroidered structures made of copper wires as current collectors for anodes. VA-CNTs were directly grown on the embroidered current collector with PECVD, in a previously optimized process [17]. No post-treatment on the VA-CNTs was applied. The electrochemical performance of the as-grown VA-CNTs on the embroidered Cu current collectors was evaluated in a half-pouch cell by means of charge/discharge cycles, rate capability, and electrochemical impedance spectroscopy (EIS) measurements. The results were compared with the state-of-art literature. This new approach provides promising possibilities in terms of three-dimensional (3D) electrode designs and the electrochemical performance of VA-CNTs, and support further progress in Li-ion batteries.

## 2. Results

### 2.1. Morphological Characteristics of the Anodes

Embroidered structures consist of a composition of metal wires on a textile fabric formed by lockstitching, a common manufacturing process in the textile industry (Figure S1). The process requires two yarns, the embroidery and the looper yarn, and a supporting fabric (which can be removed afterwards by chemical treatment). For the manufacture of the present structures, Cu metal wires were employed as embroidery and looper yarns [19].

Technical embroidery allows the achievement of 3D porous structures with metal wires of different materials and diameters. The customization of wire arrangements is possible to further optimize anode characteristics such as the specific surface area (cm^2^·g^−1^), total surface area, dimensions, and shape.

**Figure 1:**
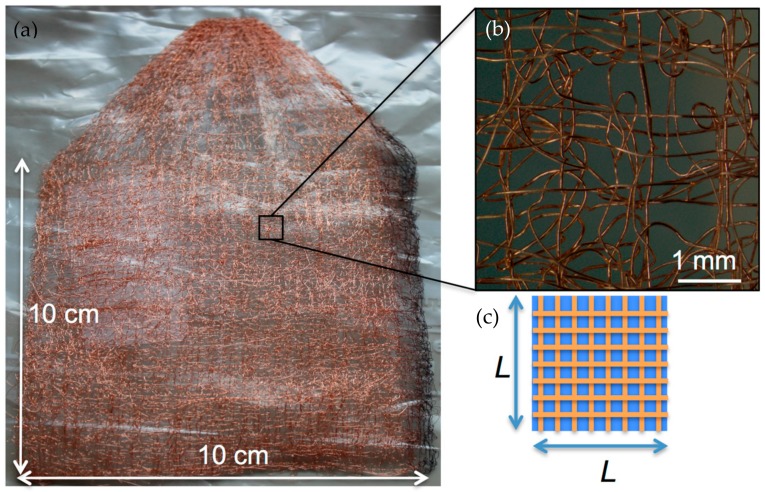
(**a**) Digital image of the Cu embroidered structure used as a current collector. (**b**) Zoomed image of a photomicrograph of the structure where front and back Cu wires are shown. The front Cu wires follow the layout illustrated in the scheme and the front wires form loops to hold the embroidered structure. (**c**) Layout of four layers with an arrangement of two horizontal and two vertical layers, with an interspacing of 0.625 mm, and with a width and length of *L*.

The Cu foils employed in anodes for Li-ion batteries normally have a thickness of 9–25 μm, depending on the current density required for their applications. Their specific surface area only depends on the thickness and Cu density, and ranges between 124–45 cm^2^ g^−1^. The same occurs with the embroidered structures, whose specific surface area depends on the Cu wire diameter. Equation (1) compares the specific surface areas of a Cu foil with an embroidered structure, similar to that shown in Figure 1. The surface area of the embroidered structure (*S*_Cu embr_), *N* × π × *D* × *L*, is calculated using the diameter of the wire *D* (assuming all wires have the same diameter, *D*), the length *L*, and the number of wires *N* included in the projected area *L*^2^. The mass of the embroidered structure (*M*_Cu embr_) is obtained by multiplying the Cu density, *ρ*, with the volume of the total wires that appear in *L*^2^, *N* × π × (*D*/2)^2^ × *L*.

Thus, the same specific surface area as the foil (*S*_Cu foil_/*M*_Cu foil_) can be obtained with a wire diameter four times the thickness (*h*) of the foil (Equation (1)).

*S*_Cu foil_/*M*_Cu foil_ = *S*_Cu embr_/*M*_Cu embr_ = *L*^2^/(*ρ* × *L*^2^ × *h*) = *N* × π × *D* × *L*/(*ρ* × *N* × π × (*D*/2)^2^ × *L*) → *D* = 4*h* (1)

This simple calculation shows one of the benefits of the embroidered structures. Thicker wires can be employed to obtain the same specific surface areas as the foils, suggesting the possibility of applying larger current densities. Following similar calculations, we determined that the surface area of the embroidered structures only depends on the number of wires *N*, which can be easily increased by reducing the wire interspacing or by increasing the number of embroidered layers.

Figure 1 shows an image of the embroidered structure used as a current collector in the experiments. In this case, the front Cu wire employed for embroidery had a diameter of 100 µm and the back Cu wire had a diameter of 80 µm. The back wire followed the pattern shown in the scheme of Figure 1, which consists of four layers of vertical and horizontal wires. Wires are separated at a distance of 0.625 mm. Further details of the layout can be found in Reference [20]. The front Cu wires formed loops to hold the Cu embroidered structure. The total length of the Cu wires used during embroidery is known, and the calculated surface area is about 49 cm^2^ g^−1^, which corresponds to a total surface area 1.8 times larger than a Cu foil surface area. In future work, further embroidered layouts will be investigated with larger values of specific surface area.

**Figure 2:**
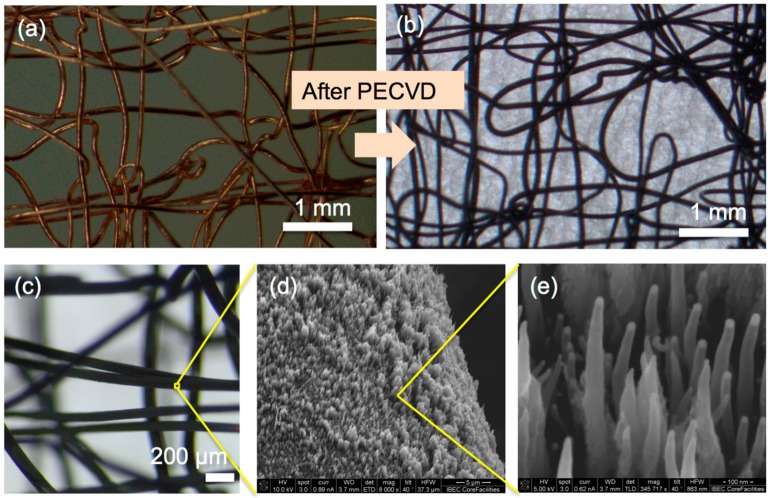
Photomicrographs of the Cu embroidered current collector (**a**) before and (**b**,**c**) after the growth of the vertically-aligned carbon nanotubes (VA-CNTs) with plasma-enhanced chemical vapor deposition (PECVD); (**d**,**e**) SEM images of the as-grown VA-CNTs. In part (**a**), the specimen was placed directly on the microscope stage and in parts (**b**,**c**), a paper was placed under the specimen.

PECVD permitted the direct growth of VA-CNTs on the embroidered Cu current collectors without the use of polymer binders (Figure 2). This allows the increase of the electroactive material available on the anode. The estimated VA-CNTs mass loading is about 0.81 mg (0.18 mg·cm^−2^), 1.8 times more than the corresponding to Cu foil of the same dimensions, as a consequence of the larger total surface area of the embroidered structures.

The scanning electron microscope (SEM) images revealed an average height and thickness of the VA-CNTs of about 10 µm and 30 nm, respectively (Figure S2). Shorter VA-CNTs appeared to have grown on the areas where the embroidered structure was not facing the plasma (Figure S3). Further investigations will be required for a detailed characterization of the effect of PECVD on the VA-CNTs morphological characteristics on embroidered structures. In addition, we cannot discard a semi-complete coverage of deposited VA-CNTs where wires crossed (Figure S4). Future work will be focused on an optimization of the embroidered layout to avoid this effect.

### 2.2. Electrochemical Performance of the Anodes

**Figure 3:**
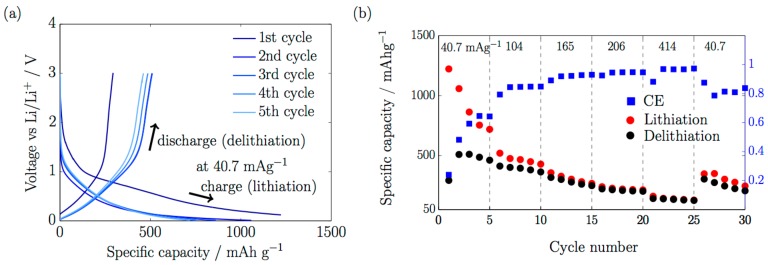
(**a**) First five cycles at 40.7 mA·g^−1^ during charge (lithiation) and discharge (delithiation); (**b**) Rate capability with cycle number during charge (red circles) and discharge (black circles), and coulombic efficiency (blue squares).

Lithiation and delithiation tests at different current densities were investigated. Figure 3a shows the first lithiation process at 40.7 mA·g^−1^ with an initial specific charge capacity of about 1222 mAh·g^−1^, and a voltage profile with a shoulder at 0.77 V. This is associated with the formation of the solid-electrolyte-interface (SEI) [21]. The voltage profile does not exhibit the typical voltage plateau at 0.8 V, in the first cycle, as reported for CNT samples with similar morphological characteristics [5,22]. This is an indication that the SEI formation is not as pronounced as in other similar CNT morphologies. The specific discharge capacity in the second cycle is also larger than reported values of about 500 mAh·g^−1^. It can be attributed to the length polydispersity of the sample, where shorter CNTs were observed on the reverse of the embroidered structures (Figure S3). Shorter CNTs show better performances than longer CNTs, since the effective diffusion of Li ions is greater within shorter lengths, as reported by Xiong et al. [8]. Further studies are required to investigate the electrochemical role of the CNT length on embroidered structures. The use of cylindrical wires, instead of planar foils, may also enhance the Li^+^ diffusion inside the VA-CNTs.

The first cycle during delithiation in Figure 3a showed smaller specific discharge capacity than the following cycles, at 293 mAh·g^−1^. This might be related to the fact that the as-grown CNTs were not purified, and unwanted species could react in the first lithiation process with the available Li^+^. However, that did not appear to have a severe consequence, since the coulombic efficiency (CE) increased during cycling, as observed in Figure 3b. The irreversible electrolyte decomposition due to the SEI formation was assumed to be completed after the first five cycles, where the coulombic efficiency (CE) increased from 24% to 79%, to finally reach 97%. After 25 cycles there was a decrease in CE to 80% and a lost in the specific capacity values at 40.7 mA·g^−1^. We cannot discard some degradation in the battery performance most probably because of atmospheric contamination. Pouch cells, in comparison with other configurations mostly found in the literature such as coin cells, are more sensitive to atmospheric contamination due to a more complicated battery assembly and sealing, but their configuration is closer to the requirements of a commercial pouch Li-ion battery in terms of battery dimensions and electrical connections. Further investigations are required to clarify this point. In spite of this, a high specific current of 414 mA·g^−1^ could be applied during cycling, indicating a good electronic conductivity of directly grown VA-CNTs on Cu embroidered structures.

**Figure 4:**
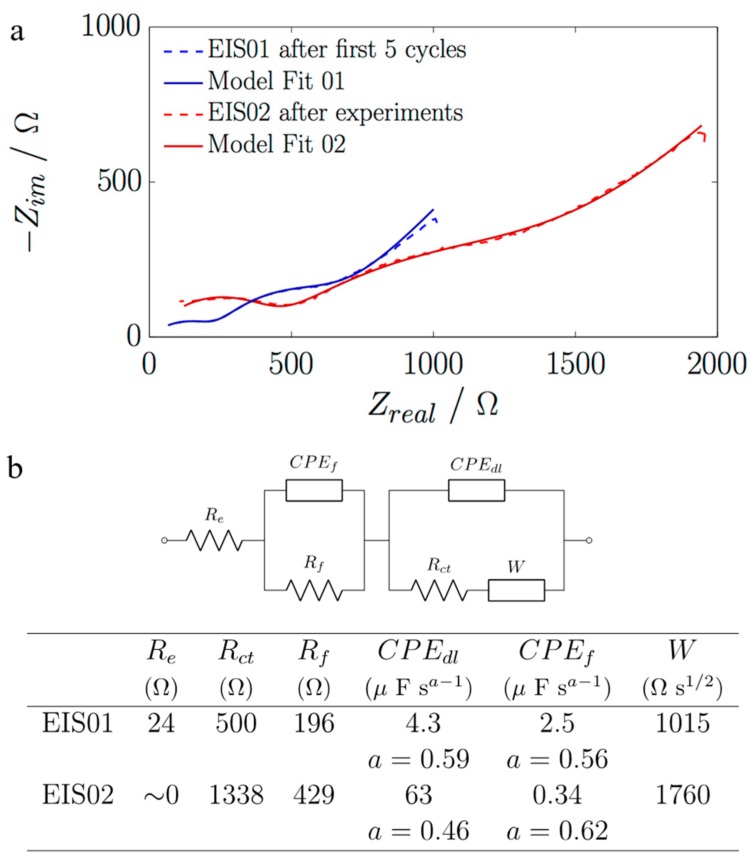
(**a**) Electrochemical impedance spectroscopy (EIS) measurements after the first five cycles and at the end of the experiments; (**b**) Corresponding equivalent circuit values from the model fit.

EIS values were investigated after the first five cycles and at the end of the experiments (Figure 4). Three clear frequency regions with two depressed semicircles were identified, and the commonly used equivalent circuit for CNTs [4] was satisfactorily applied (χ^2^ < 0.08). The 1–10 Hz region was identified with the Warburg diffusion (*W*) due to the diffusion of Li^+^ into the CNT bulk. The region from 10 Hz to 5 KHz (first depressed semicircle) corresponds to the charge-transfer resistance (*R*_ct_) and double layer capacitance behavior (*CPE*_dl_), and the region from 5 to 100 kHz is related to the resistance due to the SEI formation (*R*_f_), passivation film capacitance (*CPE*_f_), and electrolyte resistance (*R*_e_).

For the real impedance component at the lowest frequency, a range of values can be found in the literature, about 50–800 Ω, depending on the CNT morphology characteristics [4,5] or on some special treatment applied (e.g., the presence or incorporation of iron oxide particles [9,23], CNTs with open-ends [7], or CNTs with different nitrogen contents [24]). In general, our as-grown VA-CNTs showed larger values than those found in the literature, which could be attributed to the distribution of morphologies, where longer CNTs could contribute to the high EIS values, although the specific capacities obtained were better than the values found in the literature. In addition, the values of *R_ct_* and *R_f_* increased at the end of the experiments, confirming some degradation in the battery performance. Future work will be performed to clarify the relationship between EIS values and VA-CNT morphologies grown on embroidered structures.

## 3. Discussion

**Figure 5:**
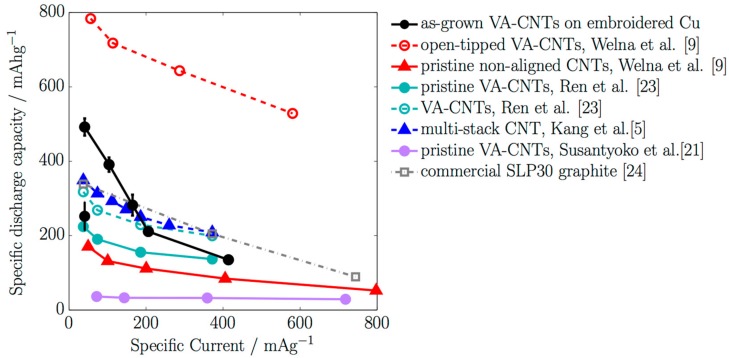
Specific discharge capacity during delithiation versus the specific current and comparison with literature values.

The specific capacities (Figure 5) and mass loadings obtained with as-grown VA-CNTs on Cu embroidered current collectors were compared with the state-of-art literature and commercial graphite [25]. The specific capacities as a function of the specific current obtained with our as-grown VA-CNTs were larger than the values found in the literature, and the approach offers more possibilities since, for instance, no post-treatment was applied.

Welna et al. [14] presented open-tipped VA-CNTs of about 8 µm in length and 77 nm in diameter, supported on a Ni thin film, and compared their behavior with non-aligned CNTs. Ren et al. [24] investigated aligned pure CNTs, with lengths larger than 15 µm and diameters of about 40–50 nm, with different Ni contents, and compared them. The mass loading was about 0.45–0.55 mg·cm^−2^. Kang et al. [4] studied free-standing multi-stacks prepared with non-aligned CNTs of about 50 µm in diameter. The samples were directly grown on Cu meshes, which were afterwards dissolved to obtain the free-standing samples. The mass loading was especially high, more than 2.72 mg·cm^−2^ depending on the sample morphology. Susantyoko et al. [22] investigated the effects of depositing Ge on VA-CNTs. Pristine VA-CNTs were used for comparison. The mass loading was about 0.10 mg cm^−2^. The mass loading of our samples was larger than the mass loading from other VA-CNTs, but can be further improved by increasing the surface area of the current collectors with the optimization of the embroidered designs.

The increase in the specific capacity of the as-grown VA-CNTs on Cu embroidered current collectors could be related to the following:

(1) A better performance of short CNTs (200 nm) compared to longer CNTs (5 µm) as described in [5]. They reached values of about 600 mAh·g^−1^ at 25 mA·g^−1^, which are still lower than our specific capacities. However, those samples were not VA-CNTs.
(2) A higher Li ion diffusion and intercalation on the CNTs. That was already observed in VA-CNTs, but the cylindrical geometry of the embroidered Cu current collectors might have helped to increase the Li^+^ diffusion and intercalation, and enhance the performance of as-grown VA-CNTs.
(3) Other factors not considered, such as the role of the metal particles on the VA-CNTs, which could also react with the Li^+^. However that effect was not observed on the voltage profiles, where larger voltages should have been observed during lithiation and delithiation.

However, in our as-grown VA-CNTs the decreasing rate of the specific capacities with the specific currents was more abrupt than the tendency from other samples plotted in Figure 5, which might be attributed to the atmospheric contamination, but further investigations will be required.

## 4. Materials and Methods 

Cu wires were embroidered on a polyester (PES) fabric using 80 and 100 µm Cu wires as embroidery and looper yarns, respectively. The PES fabric was dissolved with a 1 M NaOH solution in ethanol (96%) at 60 °C for 8 h in a water bath and then cooled down to room temperature for 14 h. The embroidered layout consisted of a vertical and horizontal arrangement of 80 µm Cu wires with a wire interspacing of 0.625 mm. Further details of the layout are described elsewhere [20].

The growth of the VA-CNTs was performed using PECVD with H_2_ plasma for 10 min on a Cu embroidered structure of 3 × 3 cm^2^ (Figure S5). Before their growth, H_2_ plasma was used to remove the native copper oxide. The plasma conditions were: 20 sccm H_2_ flow, 20 Pa, 100 W direct current (DC)-pulsed power (MKS ENI RPG-50, München, Germany), 100 kHz frequency, and 2016 ns pulse width for a duration of 10 min. Pulsed DC magnetron sputtering of Al_2_O_3_/Ti/Ni (300/50/20 nm thick) diffusion barrier was performed at 120 W DC-pulsed power (MKS ENI RPG-50, München, Germany), 100 kHz frequency, 2016 ns pulse width, and a working pressure of 1 Pa. The diffusion barrier warrants the nucleation of the iron catalyst necessary for proper CNT growth. Further details about the CNT PECVD conditions, and results concerning Raman spectroscopy and X-ray photoelectron spectroscopy are described in References [17,26]. SEM was performed using both JEOL JSM-701F (Tokyo, Japan) and JEOL JSM-7100F microscopes (Tokyo, Japan).

The pouch cell assembly (Figure 4b) was done in an Ar glovebox using a Li foil (Sigma-Aldrich, Steinheim, Germany) as anode and reference electrode, and Celgard^®^ 2400 as a separator membrane (samples courtesy of Celgard^®^, Charlotte, CA, USA). The electrolyte was composed of 1 M LiPF_6_ in 1:1 *v*/*v* ethylene carbonate and diethyl carbonate (EC/DEC) (Sigma-Aldrich Chemie, Steinheim, Germany). The cells were galvanostatically charged/discharged at different specific currents, and EIS was performed after full charge/discharge cycles (at 5 mV amplitude from 1 Hz to 1 MHz) using a SP-150 Bio-Logic system (Seyssinet-Pariset, France) with EC-Lab software (Seyssinet-Pariset, France).

## 5. Conclusions

Anodes consisting of VA-CNTs directly grown on embroidered Cu wire current collectors, with PECVD, were investigated. No post-treatment was applied. The as-grown VA-CNTs exhibited larger specific capacities compared to literature values for commercial graphite and similar VA-CNTs.

The superior electrochemical performance may be attributed to an enhancement of the Li^+^ diffusion inside the VA-CNTs because of two reasons: (1) shorter VA-CNTs were present in the areas not facing the plasma, and this facilitated Li-ion diffusion; and/or (2) there was a radial growth of the VA-CNTs around the Cu wires, and that allowed larger spaces between VA-CNTs at the outer end as compared to the base end. Further investigations are required to verify these reasons.

The employment of embroidered Cu current collectors in comparison to Cu foils offers the following advantages: higher surface area than Cu foils for the same mass; greater electronic conductivity, because the embroidered structure is composed of wires, for the same specific surface area, as wires are thicker than foils; and greater mechanical stability due to the 3D structure.

The approach offers new avenues for the further development of 3D porous electrode concepts. Additional embroidered designs can be explored in terms of geometric shapes and wire arrangement to further improve the performance of the studied VA-CNT anodes.

## Supplementary Materials

The following are available online at http://www.mdpi.com/2079-4991/7/12/438/s1, Figure S1: Schematic drawing of an embroidered structure with a layout of one layer. Cu wires are used as embroidery and looper yarns. (a) Front view showing the arrangement of one layer with vertical wires; (b) Cross-section showing the lockstitching technique, and the arrangement of the embroidery and looper yarns. Figure S2: (a) SEM image showing the Cu wire used for embroidery with a diameter of 80 µm; (b) SEM image of the Cu wires with VA-CNTs. The final diameter is about 100 µm, indicating an average VA-CNT length of about 10 µm. Figure S3: SEM images of the Cu wire with VA-CNTs from different areas: (a) front; (b) side; and (c) backside with respect to the plasma. CNTs showed a preferential growth in the vertical direction. Shorter VA-CNTs were observed in the backside. Figure S4: Photomicrograph of the embroidered structure after the growth of VA-CNTs. Some areas where Cu wires crossed (marked with a white rectangle) seem to be semi-completely covered by VA-CNTs. Figure S5: (a) Growth of VA-CNTs on 3 × 3 cm^2^ embroidered Cu current collectors; (b) Preparation of a half-pouch cell of 1.5 × 3 cm^2^.
