# Supplementary material for: New Three-Dimensional Porous Electrode Concept: Vertically-Aligned Carbon Nanotubes Directly Grown on Embroidered Copper Structures
Source: Nanomaterials (Basel). 2017 Dec 11;7(12):438. doi: 10.3390/nano7120438 (PMC5746928; doi:10.3390/nano7120438)
Supplement: Supplementary file 1 [file nanomaterials-07-00438-s001.pdf]

Supplementary Material:

New three-dimensional porous electrode concept: vertically-aligned carbon nanotubes directly growth on embroidered copper structures

Noemí Aguiló-Aguayo [1,*], Roger Amade [2,3], Shahzad Hussain [2], Enric Bertran [2,3] and Thomas Bechtold [1]

1 Research Institute of Textile Chemistry and Textile Physics, University of Innsbruck, Hoechsterstrasse 73, Dornbirn 6850, Austria; thomas.bechtold@uibk.ac.at

2 FEMAN Group, Departament de Física Aplicada, Universitat de Barcelona, c/Martí i Franquès 1, 08028 Barcelona, Catalonia, Spain; r.amade@ub.edu (R.A.); sha.awan@hotmail.com (S.H.); ebertran@ub.edu (E.B.)

3 Institute of Nanoscience and Nanotechnology (IN2UB), Universitat de Barcelona, c/Martí i Franquès 1, 08028 Barcelona, Catalonia, Spain

* Correspondence: noemi.aguilo-aguayo@uibk.ac.at or noeaguilo@gmail.com or textilchemie@uibk.ac.at; Tel.: +43-5572-28533-583

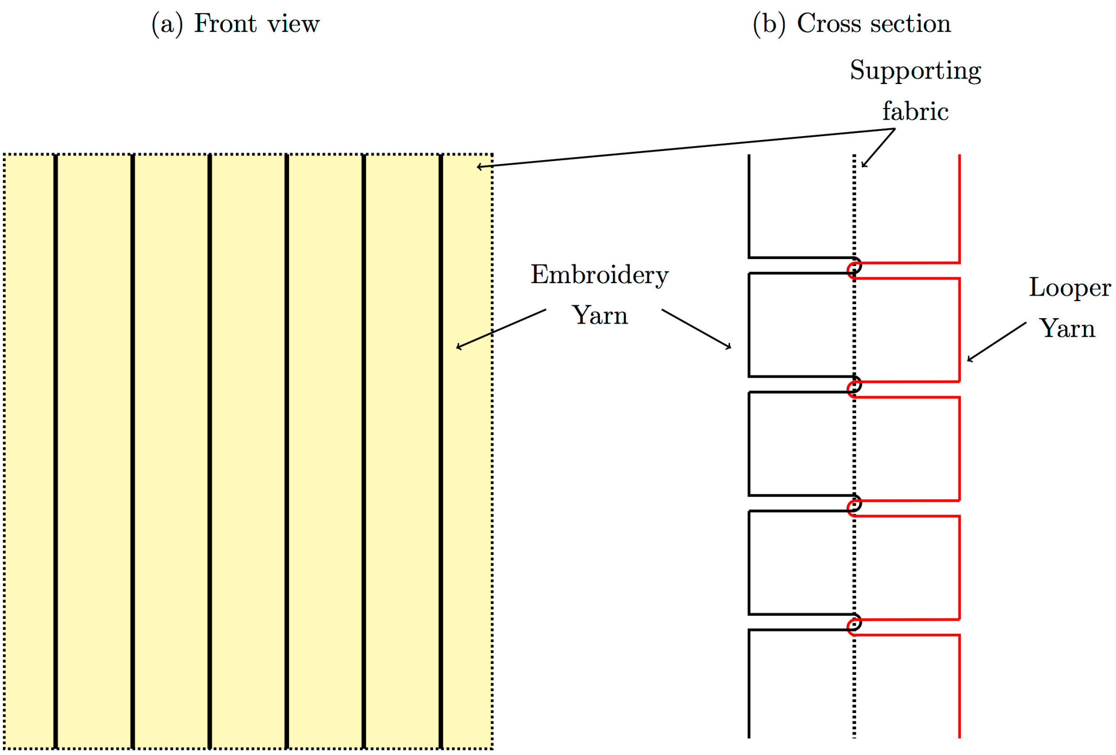

Figure S1. Schematic drawing of an embroidered structure with a layout of one layer. Cu wires are used as embroidery and looper yarns. (**a**) Front view showing the arrangement of one layer with vertical wires; (**b**) Cross section showing the lockstitching technique, and the arrangement of the embroidery and looper yarns.

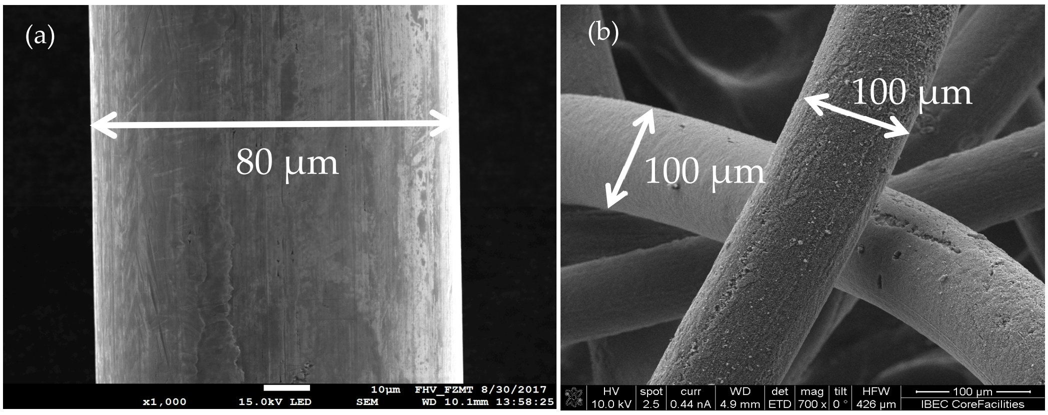

Figure S2. (**a**) SEM image showing the Cu wire used for embroidery with a diameter of 80 μm; (**b**) SEM image of the Cu wires with VA-CNTs. The final diameter is about 100 μm, indicating an average VA-CNT length of about 10 μm.

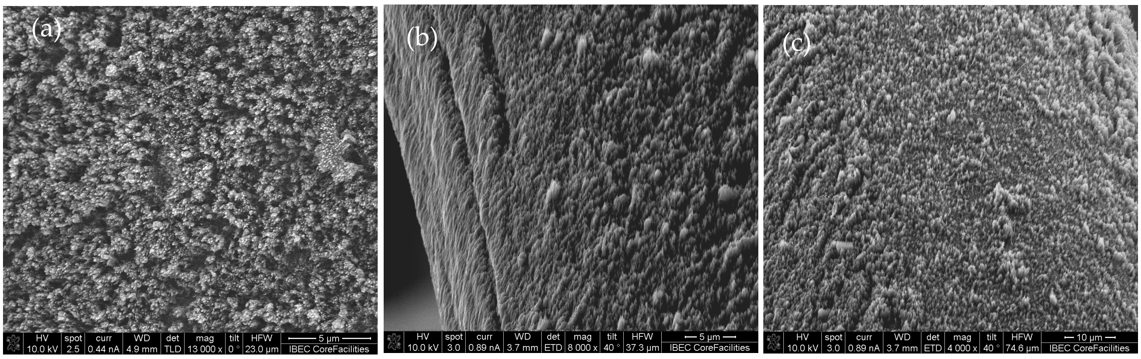

Figure S3. SEM images of the Cu wire with VA-CNTs from different areas: (**a**) front; (**b**) side and (**c**) backside with respect to the plasma. CNTs showed a preferential growth in the vertical direction. Shorter VA-CNTs were observed in the backside.

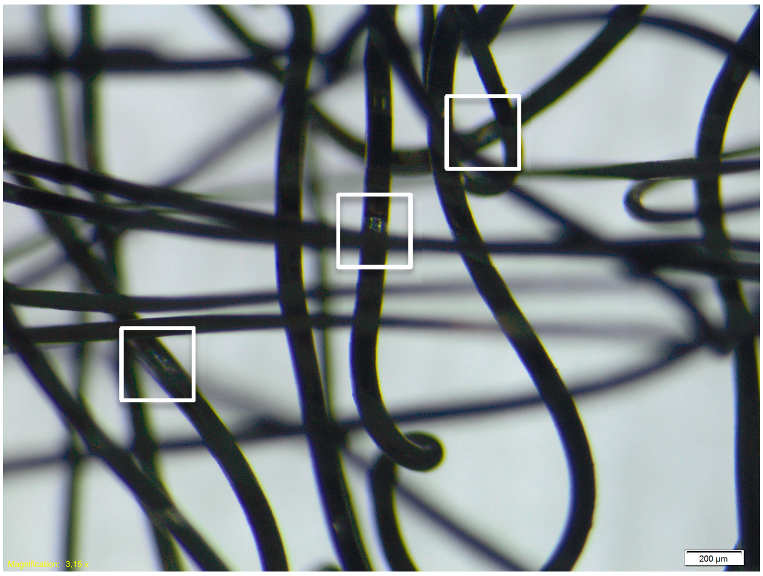

Figure S4. Photomicrograph of the embroidered structure after the growth of VA-CNTs. Some areas where Cu wires crossed (marked with a white rectangle) seem to be semi-completely covered by VA-CNTs.

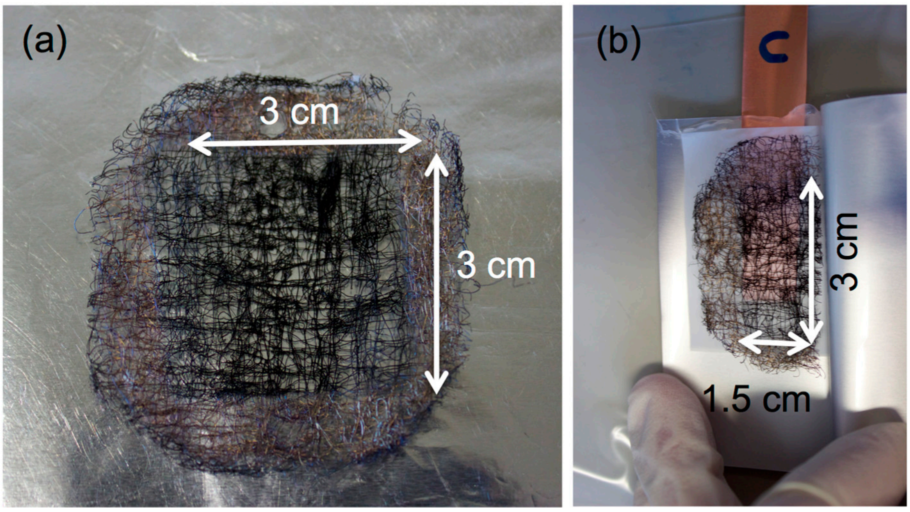

Figure S5. (**a**) Growth of VA-CNTs on 3 × 3 cm² embroidered Cu current collectors; (**b**) Preparation of a half-pouch cell of 1.5x3 cm².
